# Soil microbial diversity: A key factor in pathogen suppression and inoculant performance

**DOI:** 10.1016/j.geoderma.2025.117444

**Published:** 2025-08

**Authors:** Caroline Sayuri Nishisaka, Hélio Danilo Quevedo, João Paulo Ventura, Fernando Dini Andreote, Tim H. Mauchline, Rodrigo Mendes

**Affiliations:** a Embrapa Environment, Jaguariúna, SP, Brazil; b College of Agriculture “Luiz de Queiroz”, University of São Paulo, Piracicaba, SP, Brazil; c Sustainable Soils and Crops, Rothamsted Research, Harpenden, Hertfordshire, UK

**Keywords:** Dilution-to-extinction, Rhizosphere microbiome, *Pseudomonas inefficax* strain CMAA1741, *Bipolaris sorokiniana*, Chitinophagaceae, Soil-borne pathogen

## Abstract

Soil microbial diversity plays a crucial role in plant health, influencing pathogen suppression and biocontrol efficacy. This study investigated how soil microbial diversity modulates interactions between the pathogen *Bipolaris sorokiniana* and the biocontrol bacterium *Pseudomonas inefficax* in the wheat rhizosphere. Using a dilution-to-extinction method, we established five soil microbial diversity levels: natural soil, dilutions at 10^-1^, 10^-3^, 10^-6^, and fully autoclaved soil. This gradient allowed us to evaluate disease severity, plant growth, and rhizosphere microbiome shifts. Inoculation with *Pseudomonas inefficax* significantly reduced disease severity caused by *Bipolaris sorokiniana*, particularly in low-diversity soils, emphasizing the effectiveness of *P. inefficax* in these simplified environments where microbial competition is reduced. Despite higher pathogen abundance in low-diversity soils, *P. inefficax* effectively mitigated disease severity, likely through direct antagonistic activity. Alpha diversity indices confirmed a reduction in microbial diversity across the gradient, while beta diversity analyses revealed distinct shifts among treatments. Although *Chitinophaga*, *Pseudomonas* and *Dyadobacter* were significantly enriched in natural soils with inoculation of the *P. inefficax*, statistically significant disease suppression was not observed under these higher-diversity conditions. On the other hand, in low-diverse soils (autoclaved soil), where disease is suppressed with *P. inefficax* inoculation, *Fluviicola* showed a significant enrichment when compared with the treatment inoculated only with the pathogen, suggesting that this bacterial taxon can play a role in disease suppression along with the inoculant. These findings underscore the critical role of the soil microbial diversity in shaping the success of biocontrol interventions.

## Highlights

• Low soil microbial diversity correlates with increased disease severity in wheat.
• Inoculation with *Pseudomonas inefficax* reduces disease, especially in soils with low microbial diversity.
• High microbial diversity may hinder inoculant performance due to microbial competition.
• *Fluviicola* enrichment in the rhizosphere correlates with suppression of *Bipolaris sorokiniana*.

## 1 Introduction

The ability of a pathogen to infect a plant often depends on the presence of specific resistance genes in the host (Kushalappa et al., 2016, Fernandez-Gutierrez and Gutierrez-Gonzalez, 2021). While such genetic resistance is commonly effective against airborne pathogens, it is less frequent for soil-borne diseases (McDonald et al., 2018, Singh, 2017). This suggests that plants may have evolved alternative defense strategies belowground, such as recruiting beneficial microorganisms to the rhizosphere to help suppress pathogens (Cook et al., 1995, Mendes et al., 2011, Chapelle et al., 2016, Carrión et al., 2019).

The rhizosphere, the narrow soil zone around plant roots, is a dynamic environment where root exudates, soil, and microbial communities interact (Hiltner, 1904, Sasse et al., 2018). Through exudation of compounds like sugars, amino acids, and secondary metabolites, plants can shape the microbial community, favoring organisms that suppress pathogens by outcompeting them or producing antimicrobial substances (Raaijmakers and Mazzola, 2012, Zhalnina et al., 2018). These interactions can also activate plant immune responses, further strengthening defenses against root infections (Trivedi et al., 2020, Chapelle et al., 2016). Moreover, studies have highlighted that the soil microbiome composition plays a crucial role in modulating disease suppression, with microbial diversity being key to enhancing plant resilience (Costa et al., 2023, Mendes et al., 2019).

While microbiome recruitment can occur naturally (Mendes et al., 2013), microbial inoculation has emerged as a promising strategy to enhance plant health (Cunha et al., 2024), particularly in agricultural systems where soil-borne pathogens threaten crop productivity (O'Callaghan et al., 2021, Kashyap et al., 2023). Given the growing need for sustainable alternatives to chemical pesticides, microbial inoculation offers an environmentally friendly solution that can be tailored to specific soil microbiomes, thereby optimizing biocontrol efficacy (Kashyap et al., 2023).

In this context, several studies underscore the crucial role of the rhizosphere microbiome in protecting plants from pathogens (Mallon et al., 2015, Chapelle et al., 2016, Mendes et al., 2019, Wei et al., 2015) and highlight the importance of soil microbial composition and diversity in influencing pathogen success (Wang et al., 2019, Hu et al., 2020). However, less is known about how soil and rhizosphere microbial diversity affect the effectiveness of beneficial inoculants. Like pathogens, these inoculants must navigate complex microbial interactions, establish niches, and compete for resources to exert their beneficial effects.

This study aimed to explore the interactions between the soil and rhizosphere microbiome and soil-borne pathogens, with a particular focus on how differences in soil microbial diversity influence disease suppression. To test the hypothesis that the establishment and success of a soil pathogen − or a beneficial inoculant − is conditioned by the diversity of the soil microbiome, we employed a dilution-to-extinction approach combined with 16S rRNA gene and ITS region sequencing. For the bioassay, we used the wheat pathogen *Bipolaris sorokiniana,* which causes brown spot and root rot, severely impacting plant health by disrupting photosynthesis and nutrient uptake (McDonald et al., 2018, Singh, 2017). The antagonist bacterium *Pseudomonas inefficax* (strain CMAA1741) was used as a beneficial inoculant. Our findings provide critical insights into the role of soil microbial diversity in shaping pathogen dynamics and the success of beneficial inoculants, highlighting the potential for microbiome-based approaches in developing sustainable plant protection strategies.

## 2 Material and methods

### 2.1 Physical-chemical analysis of the soil

The soil used in the experiment was collected from the experimental field of Embrapa Environment (22°72′77.11″ N and 47°01′76.85″ W) and was classified as an Oxisol. Authorization for field soil sampling was registered with the Brazilian National System for the Management of Genetic Heritage and Associated Traditional Knowledge (SISGen) under number A5EB05F.

Soil pH was measured by mixing 10 cm^3^ of air-dried soil with 25 mL of 0.01 mol L^-1^ CaCl_2_ solution. The mixture was mechanically agitated for 1 h, after which the pH was determined using a calibrated pH electrode. Organic carbon content was estimated by wet oxidation with potassium dichromate and concentrated sulfuric acid, followed by titration with ferrous ammonium sulfate, adapted from Walkley and Black (1934). Phosphorus (P) concentration was determined using the molybdenum blue colorimetric method, with absorbance measured at 882 nm according with Murphy and Riley (1962). Exchangeable Ca^2+^, Mg^2+^, K^+^, and Na^+^ were extracted with 1 N ammonium acetate (pH 7.0) and quantified by atomic absorption spectrophotometry, using lanthanum to eliminate interferences and standard curves for calibration (USDA, 1967). Exchangeable acidity (H^+^ + Al^3+^) was extracted with 1 N KCl and determined by titration with 0.1 N NaOH using phenolphthalein as an indicator. A blank was also included for correction. Acidity was calculated as the difference in NaOH volume between sample and blank (Chernov, 1947). Available sulfate was extracted by boiling 5 g of soil with 6 N HCl for 30 min, followed by dilution, filtration, and spectrophotometric measurement at 420 nm, according to Williams and Steinbergs (1959). Available boron (B) in soil was extracted using a 1.25 g L^-1^ barium chloride solution and microwave heating (700 W), following Abreu et al. (1994). Quantification was performed by UV–Vis spectrophotometry at 420 nm using azomethine-H as the colorimetric reagent. Micronutrients (Cu, Fe, Mn, Zn) and potentially toxic elements (Cd, Cr, Ni, Pb) were extracted using a DTPA solution (diethylenetriaminepentaacetic acid, pH 7.3) and quantified by ICP-AES (Abreu et al., 1997). Exchangeable bases (Ca^2+^, Mg^2+^, K^+^, and Na^+^) were extracted using 1 N ammonium acetate at pH 7.0. Calcium and magnesium were quantified by atomic absorption spectrophotometry, while potassium and sodium were determined by flame photometry. The sum of these cations was calculated as the Sum of bases (SB), expressed in mmolc dm^−3^. Total nitrogen was determined by the Kjeldahl method. Soil samples were digested with concentrated H_2_SO_4_ in the presence of K_2_SO_4_ and catalytic agents (Cu, Se), and NH_4_^+^ was quantified by steam distillation followed by titration with standardized H_2_SO_4_. Electrical conductivity (EC) was measured in a 1:1 soil-to-water suspension using a conductivity meter, in accordance with USDA (1954) protocol.

Soil texture was determined using the pipette method, with particle size fractions defined as clay (<0.002 mm), silt (0.002–0.053 mm), and sand (0.053–2.00 mm) (Salazar et al., 2020). Soil bulk density was determined using a volumetric ring method, where a known soil volume was collected using a metal cylinder (ring), dried at 105 °C, and weighed. The bulk density was calculated as the ratio of oven-dried soil mass (g) to its volume (cm^3^). Field capacity (FC) was estimated by saturating soil samples in a tension table and allowing free drainage for 2 to 3 days, after which the moisture retained at −10 kPa tension was considered as FC. Permanent wilting point (PWP) was determined by equilibrating soil samples in a pressure plate extractor at −1,500 kPa. In both cases, soil moisture was quantified gravimetrically by drying the samples at 105 °C. These procedures follow standard protocols for assessing chemical and physical characteristics in tropical soils, as established by the Soil Fertility Laboratory of the Agronomical Institute of Campinas (IAC, Campinas, Brazil).

### 2.2 The dilution to extinction method

The soil collected was dried and sieved using a 2 mm sieve. The soil was autoclaved for four cycles at 120 °C for 60 min each. The dilution-to-extinction method (Hol et al., 2010) was employed to obtain soils with the microbial diversity gradient used in the bioassays. An initial soil inoculum suspension was prepared by mixing 450 g of natural soil (not autoclaved) with 900 mL of sterilized deionized water. Serial dilutions of the natural inoculum were then generated from the stock suspension. A gradient of three diversity levels, 10^-1^, 10^-3^, and 10^-6^, was created by adding 40 mL of the serially diluted inoculum to pots containing 200 g of autoclaved soil. Two additional treatments were added, natural soil (non-autoclaved) and autoclaved soil, resulting in five soils harboring distinct microbial diversities (Nishisaka et al., 2024a). After establishing the soil diversity gradient, soils were irrigated weekly with 40 mL of autoclaved deionized water, maintaining approximately 50 % of field capacity. The pots were incubated for 12 weeks to promote the colonization and stabilization of microbial communities before starting the experiment (Philippot et al., 2013).

### 2.3 *Pseudomonas inefficax* strain CMAA1741 inoculum preparation and seed inoculation

The bacterial strain *Pseudomonas inefficax* CMAA1741 (Nishisaka et al., 2024b) was selected for its antagonistic activity against *Bipolaris sorokiniana* and its ability to promote wheat growth. It was originally isolated from the rhizosphere of the landrace wheat Karakilcik (Rossmann et al., 2020).

CMAA1741 strains was initially cultivated on glucose yeast (GY) agar, supplemented with FeSO_4_H_2_O, MgSO_4_, and K_2_HPO_4_. After 48 h of growth, colonies were streaked onto Tryptic Soy Agar (TSA) plates to monitor potential contamination. Isolated colonies were subsequently cultured in Tryptic Soy Broth (TSB) media under 160 rpm agitation at 28 °C for 24 to 48 h (Nishisaka et al., 2024b). Following this, the grown bacteria underwent centrifugation at 8,000 *g* for 5 min. The resulting bacterial pellet was then thoroughly homogenized after being diluted in 20 mL of 0.85 % saline solution.

The inoculum concentration was adjusted to 10^8^ cells mL^−1^ in saline solution for seed inoculation. The concentration was verified using a UV spectrophotometer by measuring the optical density (OD) of the bacterial resuspension at 550 nm (OD_550_), with an OD_550_ of 0.1 corresponding to a concentration of 10^8^ cells mL^−1^. To achieve this, seeds were previously disinfected with 0.5 % hypochlorite and 70 % ethanol solutions. Subsequently, 20 g of wheat seeds were immersed in the bacterial suspension, while the control treatment involved immersion in distilled autoclaved water, followed by agitation at 160 rpm for 1 h. For treatments involving the bacteria, seed inoculation, and boost dose, 1 mL of *Pseudomonas inefficax* strain CMAA1741 suspension at 10^8^ cells mL^−1^ was applied to the seeds before sowing and to the plants 13 days after sowing.

### 2.4 Bipolaris sorokiniana inoculum preparation

The fungus *Bipolaris sorokiniana* was initially cultured on PDA media and incubated at 23 °C for 7 to 15 days. Following this, 10 mL of an 0.8 % Tween solution was added to all plates, and fungal cells were collected. The resulting fungal suspension was then thoroughly mixed for subsequent spore counting in a Neubauer chamber. For plant inoculation, 1 mL of fungal suspension with 10^4^ spores per mL of 0.8 % Tween solution was applied to the base of each plant stem. The pathogen was inoculated 10 days after sowing the seeds, which was 3 days before the bacterial boost dose.

### 2.5 Plant bioassay and experimental design

Five soils contrasting in microbial diversity were obtained using the dilution to extinction method, including natural soil, soil diluted to 10^-1^, 10^-3^, 10^-6^, and autoclaved soil. Soil dilutions were applied to the following systems: (i) bulk soil, (ii) plant (control), (iii) plant inoculated with *P. inefficax* strain CMAA1741 (antagonist), (iv) Plant inoculated *with Bipolaris sorokiniana* (pathogen), and (v) plant inoculated with both, the antagonist and the pathogen. The bioassay was performed in a completely randomized design, with 25 treatments and 5 repetitions, totaling 125 posts.

Wheat seeds from the cultivar BRS Guamirim, which is susceptible to *B. sorokiniana,* were treated with a bacterial suspension or autoclaved distilled water (control) before being planted in 200 g of soil. The study took place in a controlled environment at 21 ± 2°C, maintaining a 12-h light/12-h dark photoperiod. Soil moisture levels were adjusted considering the plant development phases, varying between 10 % and 20 % (v/w) (Costa et al., 2023). Plants had their height and disease severity index (DSI%) (Bateman et al., 2004, McMillan et al., 2014) assessed 35 days after emergence (DAE).

Microbiome analysis was performed after plant cultivation, coinciding with the time disease symptoms were scored. Within each soil dilution, we included a bulk soil treatment (no plant) that served as the baseline reference for the initial microbial community under each diversity level. All other treatments (plants inoculated with the pathogen, and/or antagonist) were compared against this bulk soil, allowing us to evaluate how microbial diversity shifted in response to biotic interactions.

### 2.6 Disease severity index calculation

Disease severity was assessed 35 days after wheat planting, coinciding with the onset of *Bipolaris sorokiniana* infection, which typically occurs during the early stages of wheat development (Al-Sadi, 2021). The proportion of infected plants was assessed and categorized into four groups: asymptomatic (0 = plants without symptoms), mild symptoms (1 = infected plants exhibiting a slight dark lesion solely on the cotyledon leaf), moderate symptoms (2 = infected plants displaying dark or reddish moderate symptoms on the stem), and severe symptoms (3 = infected plants showing dark, severe symptoms on the stem above the first leaf, and dead plants) (Costa et al., 2023). Subsequently, after categorizing the plants in each group, the Disease Severity Index (DSI) for each pot was calculated based on the method adapted from McMillan et al. (2014). The DSI for each pot was determined using the formula: (1 x percentage of plants scored 1) + (2 x percentage of plants scored 2) + (3 x percentage of plants scored 3), divided by the total number of categories (3); with the maximum DSI being 100 % (Costa et al., 2023).

### 2.7 Plant height and biomass evaluation

Plant heights were measured from the base of the plant to the tip of the uppermost leaf. The roots were washed and subsequently pruned, in which the shoot and root parts were separated. The shoot and roots were placed separately in paper bags and kept in an oven at 65 °C for 7 days. After that, the bags containing the dry parts were weighed, and the estimated weight of the dry biomass for each treatment was obtained.

### 2.8 DNA extraction and Quantitative Polymerase Chain Reaction (qPCR)

For rhizosphere soil sampling, the complete root system was carefully extracted from the pots, with the roots gently shaken to remove any loose soil. The soil remaining attached to the roots, known as rhizosphere soil, was then collected, transferred into 1.5 mL microtubes, and stored with the bulk soil samples at −20 °C for subsequent analysis (Rossmann et al., 2020). Genomic material extraction was performed using the DNeasy PowerSoil® Kit (QIAGEN catalog #12888–50) following the manufacturer's instructions. Quality and quantity assessments were conducted using a NanoDrop® ND-2000 Spectrophotometer and QUBIT® 2.0 (Thermo Fisher Scientific, Wilmington, DE, USA), respectively.

Quantitative polymerase chain reaction (qPCR) was employed for the quantification of the *Bipolaris sorokiniana*-specific *cos*A gene, utilizing the primer pair CosA_F_519 (5′ TCAAGCTGACCAAATCACCTTC 3′) and CosA_R_248 (AATGTCGAGCTTGCCAAAGT 3′) (Matusinsky et al., 2010, Horne, 2015). *P. inefficax* CMAA1741 was not specifically quantified by qPCR; instead, only total bacterial abundance was measured. Then, for bacterial community quantification, the 16S rRNA gene was targeted using the primer pair 926F (5′ AACTCAAAGGAATTGACGG 3′) and 1062R (5′ CTCACRRCACGAGCTGAC 3′) (Lane, 1991, Allen et al., 2005).

Both gene amplification reactions had a final volume of 10 μL, comprising 5 μL of SYBR Green ROX qPCR (Thermo Fisher Scientific, Middletown, VA, USA), 1 μL of each primer (final concentration of 2 μM), 2 μL of template, and 2 μL of ultrapure water (Milli-Q). Standard curves for each gene were constructed using serial dilutions (1:10) of a known amount of each target gene. The 16S rRNA gene standard originated from the bacterium *P. inefficax* strain CMAA1741, and the *cos*A standard originated from the fungus *B. sorokiniana* strain BS0208. The standard curve was constructed in triplicate to minimize errors, with at least five points for the curve. The quantification experiments were conducted using a ViiA 7 Real-Time PCR System.

### 2.9 Metataxonomic sequencing of 16S rRNA gene and ITS region

To explore community composition patterns and treatment-induced shifts, DNA samples underwent amplicon sequencing on the Illumina MiSeq v2 platform at Argonne National Laboratory (Lemont, IL, USA), generating 2 x 150 bp reads for bacterial amplicon and 2 x 250 bp reads for fungal amplicon. The sequencing targeted the 16S rRNA gene region V4 and the ITS1 region. For the 16S rRNA gene V4 region, library construction was carried out using the primer pair 515F (5′ GTGYCAGCMGCCGCGGTAA 3′) and 806R (5′ GGACTACNVGGGTWTCTAAT 3′) (Apprill et al., 2015, Parada et al., 2015). Similarly, for the ITS1 region, the primer pair ITS1f (5′ CTTGGTCATTTAGAGGAAGTAA 3′) and ITS2 (5′ GCTGCGTTCTTCATCGATGC 3′) was used (White et al., 1990).

### 2.10 Data processing and statistical analyses

Data from plant heights, disease severity index (SDI%), dry weights of the shoot and root parts, and the number of copies of 16S rRNA and *cos*A genes were compared using Tukey analysis (p < 0.05). Such comparisons occurred between the different treatments within each dilution and between the same treatment in different soil dilutions.

For metataxonomic analysis, all reads were assembled using Dada2 version 1.21.0 (Callahan et al., 2016). The primer pair removal was carried out using Cutadapt version 3.4 (Martin, 2011). After this step, quality control procedures were implemented, and reads with low quality (Q20 or lower) were excluded. Taxonomic assignment was performed utilizing the Silva (v. 138.1) and UNITE (v. 9.0) databases (Quast et al., 2013, Yilmaz et al., 2014, Nilsson et al., 2018, Kõljalg et al., 2020, Abarenkov et al., 2022). To control for differences in library sizes, sequences were rarefied using function “rarefy()” from the vegan package (v. 2.6–10) (Oksanen et al., 2025), this procedure standardizes counts across samples in a way analogous to RNA-seq normalization, mitigating compositional-data biases without needing external scaling factors (Schloss, 2024).

For alpha diversity assessment, the Chao1 and Shannon indexes were computed. Beta diversity was evaluated using the Bray-Curtis distance, and Principal Coordinate Analysis (PCoA) was employed for visualizing the similarity matrix among different dilutions and treatments. In both analyses, rarefied and normalized data were utilized. The significance and effect size of beta diversity were determined through permutation-based analysis (PERMANOVA) using the vegan package (v. 2.6–4) with the “adonis()” function (Riggs et al., 2008). Relative abundance and Venn diagrams were prepared using phyloseq (v. 1.46.0) and ggVennDiagram (v. 1.5.2) packages, respectively (McMurdie and Holmes, 2014, Gao et al., 2021). To identify differentially abundant taxa among the treatment groups, ANOVA-Like Differential Expression analysis (ALDEx2) was conducted using the “run_aldex()” function from the microbiomeMarker package (v. 1.2.2) (Fernandes et al., 2014, Cao et al., 2022). To identify microbial taxa that best differentiate between specific treatments, Random Forest analysis and multiple linear regression were performed using the MicrobiomeAnalyst 2.0 platform (Lu et al., 2023). In parallel, a General Linear Model analysis was conducted using the MaAsLin2 (Multivariate Association with Linear Models) framework, which fits generalized linear models to identify significant associations between microbial features and metadata (Mallick et al., 2021, Lu et al., 2023).

## 3 Results

### 3.1 Soil microbiome alpha diversity gradient obtained with the dilution-to-extinction method.

As expected, Chao1 and Shannon indices indicated higher microbial diversity in natural soil, followed by the 10^-1^ to 10^-6^ dilutions, with autoclaved soil showing the lowest diversity for both bacterial and fungal communities (Fig. S1). The highest Shannon index for the bacterial community was observed in natural soil, followed by dilutions 10^-1^ and 10^-3^, with dilution 10^-6^ and autoclaved soil showing the lowest diversity (Tukey HSD test; P < 0.05). A similar pattern was observed for fungal alpha diversity, where natural soil had the highest Shannon index, followed by dilution 10^-1^, dilution 10^-3^, dilution 10^-6^ and autoclaved soil, respectively.

### 3.2 *Pseudomonas inefficax* strain CMAA1741 and *Bipolaris sorokiniana* effects on plant health

**Fig. 1:**
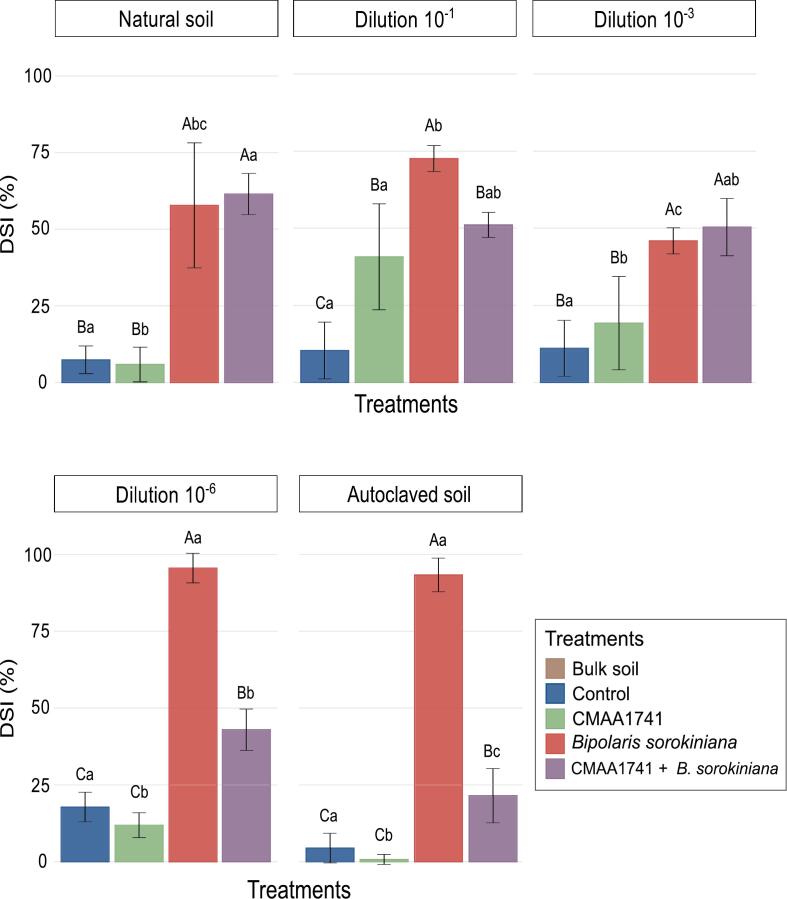
Disease severity index (DSI) including the following treatments: bulk soil (pots without plants), control (non-treated plants), CMAA1741 (plants inoculated with the antagonistic bacterium), *Bipolaris sorokiniana* (plants inoculated with the pathogen), and CMAA1741 + *B. sorokiniana* (plants inoculated with the antagonist and with the pathogen), in five different soil dilutions. Mean comparisons were conducted using the Tukey test (*P* < 0.05). Uppercase letters denote comparisons between treatments within the same soil diversity, and lowercase letters indicate comparisons of the same treatment across different soil dilutions.

Physical-chemical analysis of the natural (non-autoclaved) and autoclaved soils showed no significant differences in soil characteristics (Table S1), indicating that soil autoclaving had minimal effects on these parameters. We observed a slight increase in manganese (Mn) and sulfur (S) levels in autoclaved soils. The disease severity index (DSI%) revealed that soils with lower diversity inoculated with *Bipolaris sorokiniana* exhibited higher DSI values. However, in treatments where the antagonistic bacterium (*P. inefficax* strain CMAA1741) and *B. sorokiniana* were inoculated, the DSI progressively decreased as soil diversity declined (Fig. 1). The lowest disease severity was observed in autoclaved soil inoculated with CMAA1741, while the highest DSI occurred in both the 10^-6^ dilution and autoclaved soil inoculated solely with *B. sorokiniana* (Fig. 1).

The *cos*A gene copy number from *B. sorokiniana* remained consistent across treatments (Fig. S2), but significant differences were noted across different soil dilutions within each treatment (Fig. S2). Despite the high abundance of the *cos*A gene in the rhizosphere of the treatment inoculated with both CMAA1741 and *B. sorokiniana*, especially in less diverse soils, the lower DSI values indicated that the antagonistic bacterium effectively suppressed *B. sorokiniana* in soils with reduced diversity (Fig. 1). In less microbial-diverse soils (from the 10^-1^ dilution to autoclaved soil), a higher 16S rRNA gene copy number was found when compared to natural soil (Fig. S3).

In addition to enhancing disease suppression, inoculation with CMAA1741 significantly increased plant heights in the 10^-6^ dilution (Fig. S4). Moreover, inoculation with CMAA1741 led to a notable increase in root dry mass in natural soil compared to the combined inoculation of CMAA1741 + *B. sorokiniana* (Fig. S5).

### 3.3 Effects of *Pseudomonas inefficax* strain CMAA1741 and *Bipolaris sorokiniana* inoculation on the wheat rhizosphere bacterial community assembly

**Fig. 2:**
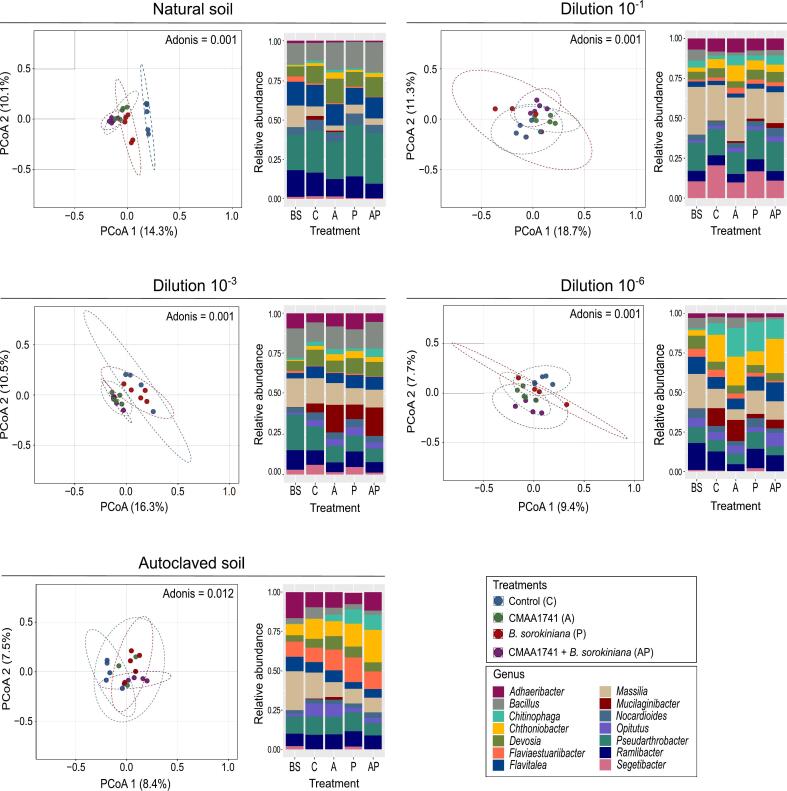
Rhizosphere bacterial community structure and composition across soil dilutions. Principal coordinate analysis (PCoA) of 16S rRNA gene amplicon in different soils diversities and treatments. Statistical pairwise comparisons were performed using the Adonis method (*P* < 0.05, permutation = 999) (Table S2). Relative abundance of bacterial genera across all treatments. BS = bulk soil, C = control (non-treated plants), A = CMAA1741 (*Pseudomonas inefficax* strain CMAA1741), P = *Bipolaris sorokiniana*, AP = CMAA1741 + *B. sorokiniana*.

Bacterial beta diversity varied significantly across treatments and soil dilutions (Adonis, *P* < 0.05) (Fig. 2). Pairwise comparisons in natural soil (Table S2) revealed that all treatments harbored distinct bacterial communities (Fig. 2). At the 10^-1^ and 10^-3^ dilutions, significant differences were observed between CMAA1741 and the control, as well as between CMAA1741 and *Bipolaris sorokiniana* treatments. At the 10^-6^ dilution, bacterial communities were distinct for most treatment comparisons (Fig. 2 and Table S2). In autoclaved soil, significant differences in bacterial communities were detected only when comparing the control with *B. sorokiniana* and the control with CMAA1741 + *B. sorokiniana* (Fig. 2 and Table S2).

Samples from wheat rhizosphere showed an increased relative abundance of *Chitinophaga* compared to bulk soil (Fig. 2). According to the differential abundance test, inoculation with CMAA1741 significantly (*P* < 0.001) enriched several microbial classes in the rhizosphere compared to the control, including *Verrucomicrobiia*, *Sumerlaeia*, *Sericytochromatia*, *Deltaproteobacteria*, *Nitrospiria*, *Nitrososphaeria*, *Gemmatimonadia*, *Blastocatellia*, *Bacteroidia*, *Bacilli*, *Alphaproteobacteria*, *Actinomycetes*, *Abditibacteriia*, and Phylum Armatimonadota (Fig. S7). Inoculation with *B. sorokiniana* significantly enriched (*P* < 0.001) the classes *Vampirovibrionophyceae*, *Thermoleophilia*, *Nitrososphaeria*, *Gemmatimonadia*, *Bacteroidia*, and *Bacilli* (Fig. S7). In the treatment where disease suppression was highly observed (CMAA1741 + *B. sorokiniana*), significant enrichment (*P* < 0.001) was noted in classes such as *Verrucomicrobiia*, *Sericytochromatia*, *Proteobacteria*, *Deltaproteobacteria*, *Nitrososphaeria*, *Holophagae*, *Oligoflexia*, *Bacteroidia*, *Bacilli*, *Alphaproteobacteria* (Fig. S7).

### 3.4 Effects of CMAA1741 and *Bipolaris sorokiniana* inoculation on the wheat rhizosphere fungal community

**Fig. 3:**
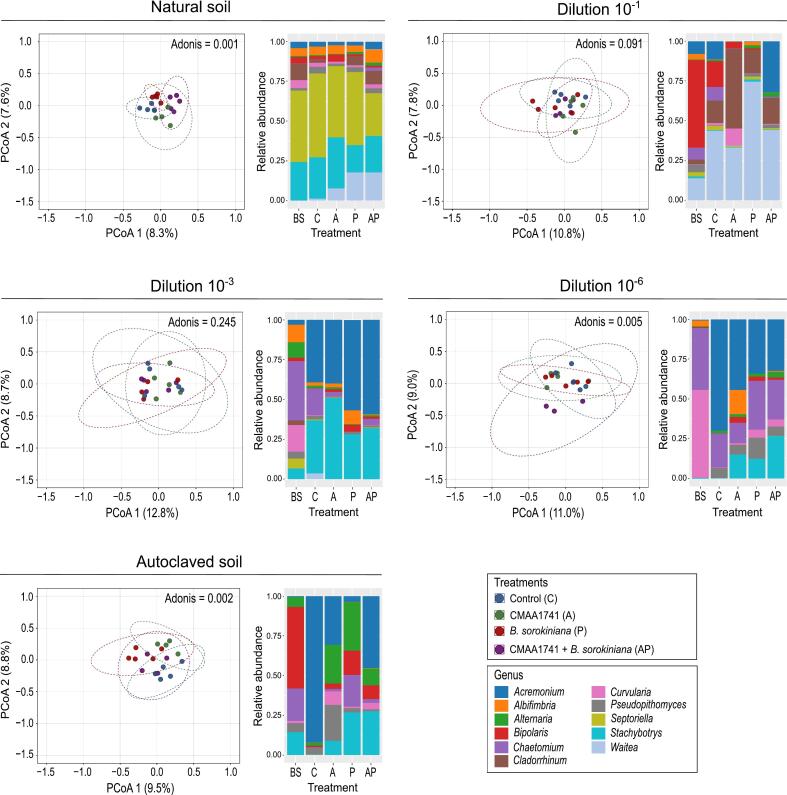
Rhizosphere fungal community structure and composition in different soil dilutions. Principal coordinate analysis (PCoA) of ITS amplicon in different soils diversities and treatments, Statistical pairwise comparisons were performed using the Adonis method (*P* < 0.05, permutation = 999) (Table S2). Relative abundance of bacterial genera across all treatments. BS = bulk soil, C = control (non-treated plants), A = CMAA1741 (*Pseudomonas inefficax* strain CMAA1741), P = *Bipolaris sorokiniana*, AP = CMAA1741 + *B. sorokiniana*.

Fungal beta diversity varied significantly across treatments in natural soil, 10^-6^ dilution, and autoclaved soil (Adonis, *P* < 0.05) (Fig. 3). Pairwise comparisons (Table S2) in natural soil revealed that all treatments harbored distinct fungal communities, except for the comparison between CMAA1741 and CMAA1741 + *Bipolaris sorokiniana* (Fig. 3). In the 10^-6^ dilution (Fig. 3), significant differences were observed between the control and CMAA1741 + *B. sorokiniana*, as well as between CMAA1741 and CMAA1741 + *B. sorokiniana* (Table S2). In autoclaved soil (Fig. 3), significant differences in fungal communities were observed in comparisons of the control with CMAA1741, the control with *B. sorokiniana*, CMAA1741 with *B. sorokiniana*, and CMAA1741 with CMAA1741 + *B. sorokiniana* (Table S2).

A reduction in the *Bipolaris* genus was noted in rhizosphere samples compared to bulk soil, while *Acremonium* increased relative to the bulk soil treatment (Fig. 3). Inoculation with CMAA1741 resulted in the enrichment of fungal classes such as *Spizellomycetes*, *Leotiomycetes*, *Kickxellomycetes*, and *Eurotiomycetes* compared to the control (*P* < 0.01) (Fig. S8). *Bipolaris sorokiniana* inoculation also enriched (*P* < 0.01) some fungal classes, including *Spizellomycetes*, *Orbiliomycetes*, *Dothideomycetes*, and *Chytridiomycetes* (Fig. S8). In the disease suppression treatment (CMAA1741 + *B. sorokiniana*), classes like *Spizellomycetes*, *Orbiliomycetes*, *Kickxellomycetes*, *Dothideomycetes*, and *Agaricomycetes* were significantly enriched (*P* < 0.01) in the wheat rhizosphere compared to the control (Fig. S8). As observed, *Dothideomycetes*, which includes the *Bipolaris* genus, was significantly enriched in treatments inoculated with the pathogen *B. sorokiniana* (Fig. S8).

### 3.5 Key microbial taxa and treatment-driven community patterns under disease suppression

**Fig. 4:**
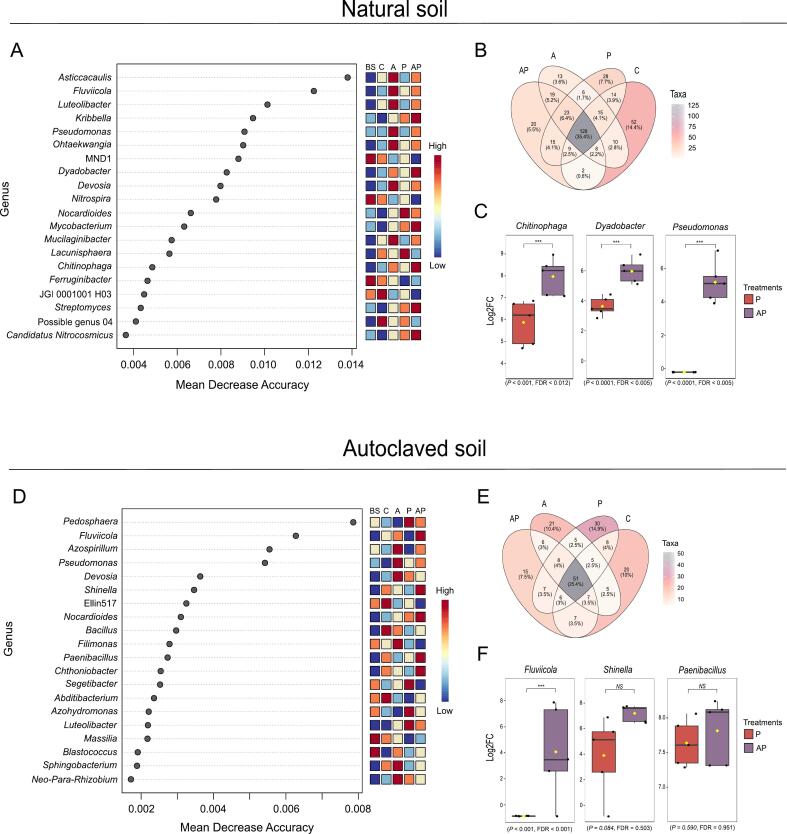
Analysis of bacterial community composition and key genera across treatments in natural and autoclaved soil. **A** Random Forest model showing the top discriminatory bacterial genera based on Mean Decrease Accuracy (MDA) (P < 0.05) in natural soil, with higher values indicating greater importance in distinguishing treatment groups. **B** Venn diagram displaying the shared and unique bacterial taxa among treatments in natural soil, considering core microbiome detection in at least 90 % of samples and a prevalence of 50 %. Overlapping regions represent taxa common to multiple treatments. **C** General Linear Model results of selected bacterial genera comparison between CMA1741 + *B. sorokiniana* and *B. sorokiniana* treatments, in natural soil, using MaAsLin2 (*P*-value cutoff 0.05, EdgeR method). **D** Random Forest model showing the top discriminatory bacterial genera based on Mean Decrease Accuracy (MDA) (P < 0.05) in autoclaved soil, with higher values indicating greater importance in distinguishing treatment groups. **E** Venn diagram displaying the shared and unique bacterial taxa among treatments in autoclaved soil, considering core microbiome detection in at least 90 % of samples and a prevalence of 50 %. **F** General Linear Model results of selected bacterial genera comparison between CMA1741 + *B. sorokiniana* and *B. sorokiniana* treatments, in autoclaved soil, using MaAsLin2 (*P*-value cutoff 0.05, EdgeR method). BS = bulk soil, C = control (non-treated plants), A = CMAA1741 (*Pseudomonas inefficax* strain CMAA1741), P = *Bipolaris sorokiniana*, AP = CMAA1741 + *B. sorokiniana*.

**Fig. 5:**
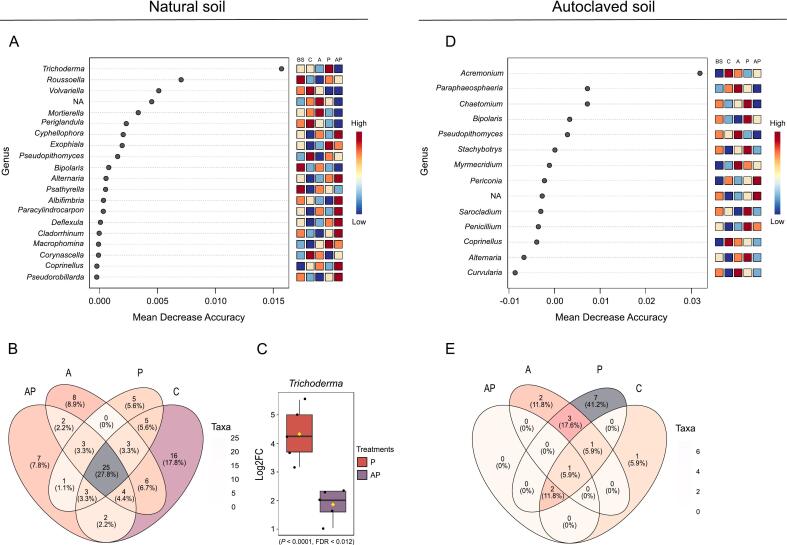
Analysis of fungal community composition and key genera across treatments in natural and autoclaved soil. **A** Random Forest model showing the top discriminatory fungal genera based on Mean Decrease Accuracy (MDA) (P < 0.05) in natural soil, with higher values indicating greater importance in distinguishing treatment groups. **B** Venn diagram displaying the shared and unique fungal taxa among treatments in natural soil, considering core microbiome detection in at least 90 % of samples and a prevalence of 50 %. Overlapping regions represent taxa common to multiple treatments. **C** General Linear Model results of selected fungal genera comparison between CMA1741 + *B. sorokiniana* and *B. sorokiniana* treatments in natural soil using MaAsLin2 (*P*-value cutoff 0.05; EdgeR method). **D** Random Forest model showing the top discriminatory fungal genera based on Mean Decrease Accuracy (MDA) (P < 0.05) in autoclaved soil, with higher values indicating greater importance in distinguishing treatment groups. **E** Venn diagram displaying the shared and unique fungal taxa among treatments in autoclaved soil, considering core microbiome detection in at least 90 % of samples and a prevalence of 50 %. BS = bulk soil, C = control (non-treated plants), A = CMAA1741 (*Pseudomonas inefficax* strain CMAA1741), P = *Bipolaris sorokiniana*, AP = CMAA1741 + *B. sorokiniana*.

To identify microbial taxa most predictive of treatment effects and disease suppression, we performed Random Forest analysis on bacterial (Fig. 4A and D) and fungal community data (Fig. 5A and D). This machine learning approach quantified the importance of individual genera through Mean Decrease Accuracy (MDA), with higher values indicating stronger discriminatory power between treatments.

The analysis revealed distinct bacterial taxa, such as *Kribbella, Dyadobacter, Mycobacterium, Chitinophaga, Streptomyces* and *Candidatus Nitrocosmicus* as key genera under treatment of CMAA1741 + *B. sorokiniana* in natural soil (Fig. 4A). Taxa overlap also showed important unique taxa related to treatment of high disease suppression (CMAA1741 + *B. sorokiniana*) in natural soil, including genera pointed by Random Forest test, such as *Streptomyces* (ASV-01461), *Chitinophaga* (ASV-01693), and *Kribbella sancticallisti* (ASV-01091) (Fig. 4B).

A Microbiome Multivariate Associations with Linear Models analysis (*P* < 0.05) was carried out by comparing the most disease suppressive treatment (CMAA1741 + *B. sorokiniana*) with the most disease conducive treatment (*B. sorokiniana*), under both, natural and autoclaved soil (Fig. 4C). In natural soil, 14 bacterial genera showed statistically significant associations with disease-suppressive treatment, in which 9 had strong positive association, including *Chitinophaga* (*P* < 0.001, FDR < 0.012), *Dyadobacter* (*P* < 0.0001, FDR < 0.005) and *Pseudomonas* (*P* < 0.0001, FDR < 0.005) genera (Fig. 4C).

Random Forest was employed to rank taxa based on their importance in discriminating between treatment groups using their relative abundances. In autoclaved soil, the CMAA1741 + *B. sorokiniana* treatment was primarily differentiated by the genera *Fluviicola*, *Shinella*, *Nocardioides*, *Paenibacillus*, and *Chthoniobacter* (Fig. 4D). Venn analysis identified treatment-exclusive taxa, including *Chthoniobacter* (ASV-00304), previously identified as high important genus in this treatment by Random Forest analysis. Plus, members of the Acidimicrobiia (ASV-00407) and Sericytochromatia (ASVs-00212, 00310, 01282) classes, and bacterial genera such as *Achromobacter* (ASV-00116), *Stenotrophomonas* (ASV-00355), *Reyranella* (ASV-00491), *Altererythrobacter* (ASV-00605), *Blastococcus* (ASV-01073), and *Edaphobaculum* (ASV-01106), along with the species *Flavisolibacter metallilatus* (ASV-00166), *Nordella oligomobilis* (ASV-00240), and *Bosea thiooxidans* (ASV-00275) (Fig. 4E) were also exclusively detected in CMAA1741 + *B. sorokiniana* treatment. The Microbiome Multivariate Associations with Linear Models analysis (P < 0.05) revealed a significant association between the *Fluviicola* genus and the CMAA1741 + *B. sorokiniana* treatment when compared to *B. sorokiniana* alone. Although *Shinella* and *Paenibacillus* showed high importance in the Random Forest analysis, their changes in abundance were not statistically significant in the linear model comparison between these two treatments.

Following the analysis of bacterial communities, Random Forest was also applied to the fungal dataset to identify taxa most strongly associated with the CMAA1741 + *B. sorokiniana* treatment in both natural and autoclaved soils. In natural soil, the model identified key fungal genera predictive of this treatment, included *Cyphellophora*, *Alternaria*, *Albifimbria*, *Paracylindrocarpon*, *Deflexula*, *Cladorrhinum*, *Coprinellus*, and *Pseudorobillarda* (Fig. 5A). Complementarily, a Venn diagram showed that *Alternaria* (ASV-0050) and *Cyphellophora* (ASV-0302) were exclusive detected in this treatment (Fig. 5B). When comparing the CMAA1741 + *B. sorokiniana* treatment with *B. sorokiniana* alone, a significant enrichment of the genus *Trichoderma* (Log2FC > X) was observed in the pathogen-only treatment compared to the double-inoculated treatment (Fig. 5C). In autoclaved soil, *Periconia* and an unidentified taxon were identified by the Random Forest model as most relevant to the CMAA1741 + *B. sorokiniana* treatment (Fig. 5D). However, no exclusive taxa were observed for this treatment in the autoclaved condition (Fig. 5E).

## 4 Discussion

Inoculation with *Bipolaris sorokiniana* significantly increased disease severity in wheat, particularly in soils with reduced microbial diversity. This indicates that the pathogen more readily colonizes the rhizosphere under conditions of limited microbial competition. These findings are consistent with previous studies suggesting that lower microbial diversity in soil creates a more conducive environment for pathogen establishment and disease progression (Caballero-Flores et al., 2023). While *Bipolaris sorokiniana* alone exacerbated disease symptoms, inoculation with *Pseudomonas inefficax* strain CMAA1741 (CMAA1741 + *B. sorokiniana*) alleviated these effects, particularly in dilutions from 10^-1^ to 10^-6^ and autoclaved soils. This reinforces the “diversity-invasibility hypothesis” (DIH) (Mallon et al., 2018), which posits that ecosystems with lower biodiversity are more susceptible to invasion due to increased niche availability and reduced competition (Van Elsas et al., 2012, Roman and Wager, 2021, Spragge et al., 2023).

We hypothesized that high rhizosphere microbial diversity may play dual, and at times opposing, roles: it can suppress pathogen colonization, yet also impede the establishment of introduced biocontrol agents through intensified microbial competition (Mendes et al., 2013, Mehrabi et al., 2016). Conversely, successful pathogen invasion can disrupt the resident microbiome and diminish diversity (Wei et al., 2019), thereby creating niches that favor the establishment of beneficial microbes such as inoculants. Thus, the impact of microbial diversity is multifaceted and highly context-dependent. Importantly, the timing of biocontrol application may be critical, as pathogen-induced shifts in community structure could facilitate subsequent colonization by biocontrol agents (Berendsen et al., 2012, Wei et al., 2019).

Beyond disease suppression, inoculation with *P. inefficax* and *B. sorokiniana* together significantly enhanced plant growth, manifested as increased height and biomass, particularly in soils exhibiting reduced microbial diversity. (Figs. S5 and S6). The plant growth–promoting effect may have been enhanced by *P. inefficax*, which likely contributed to increased plant height in natural and 10^-6^ soils and greater root dry mass in 10^-1^ soils, possibly due to traits commonly found in *Pseudomonas* species, such as IAA production and nutrient solubilization (Grosse et al., 2023, Zboralski and Filion, 2023).

Costa et al. (2023) showed that even after repeated exposure of wheat to *B. sorokiniana*, which led to clear disease suppression, the abundance of the fungal *cos*A gene remained unchanged. In our experiment, we observed a similar pattern across treatments within each soil dilution, pathogen abundance is the same despite substantial differences in disease severity between inoculated and non-inoculated soils (Fig. S2). Inoculation with CMAA1741 + *B. sorokiniana* (Fig. S2) led to effective disease suppression within a single cycle, as evidenced by the reduced Disease Severity Index (DSI) (Fig. 1), suggesting rapid colonization and pathogen inhibition, an outcome comparable to Costa et al. (2023) but achieved without the need for repeated cycles of pathogen exposure. These findings suggest that CMAA1741 suppresses disease not by reducing *B. sorokiniana* abundance, but by reinforcing rhizosphere microbiome–mediated protection, such that high pathogen presence does not translate into disease.

The soil diversity gradient significantly shaped microbial community assembly and influenced plant phenotype. The dilution approach successfully generated a gradient in bacterial and fungal diversity, as reflected in declining species richness (Chao1) and Shannon diversity indices with increasing dilution (Fig. S1). Prior research has shown that plant pathogens and their interactions with beneficial microorganisms can distinctly reshape soil microbial communities (Mendes et al., 2015, Berendsen et al., 2018). In natural soil, all pairwise comparisons showed significant differences in bacterial community composition (Table S2). This highlights the resilience of the microbial community to environmental changes, as it neither facilitated the invasion nor the establishment of CMAA1741 in the rhizosphere, ultimately leading to reduced disease suppression in plants (Mendes et al., 2013, Trivedi et al., 2020). In contrast, in autoclaved soil, no significant differences were observed between the microbial communities of *B. sorokiniana* and CMAA1741 + *B. sorokiniana* treatments (Table S2). However, the high disease suppression observed in the latter (Fig. 1) suggests that CMAA1741 alone compensated for the lack of protective diversity, likely acting through direct antagonism rather than through broader reshaping of the resident microbial community. This interpretation is supported by the fact that, aside from *Fluviicola*, no other bacterial taxa were significantly enriched in the LM analysis for this treatment.

*Chitinophaga* was significantly associated with the CMAA1741 + *B. sorokiniana* treatment in natural soil, alongside *Kribbella*, *Dyadobacter*, *Mycobacterium*, *Streptomyces*, and *Candidatus Nitrocosmicus*. However, it is important to note that disease suppression under this condition was not statistically significant (Fig. 1). *Chitinophaga* genus is noteworthy for its antifungal metabolite production and antagonistic activity, which contribute to disease suppressiveness (Garbeva et al., 2011, Li et al., 2014, Chapelle et al., 2016, Carrión et al., 2019, Deng et al., 2020). Besides, Costa et al. (2023) reported an increased abundance of Chitinophagaceae family following repeated exposure of a susceptible wheat cultivar to *B. sorokiniana*, which was associated with reduced disease severity after five successive growth cycles. In our study, we used the same wheat cultivar (BRS Guamirim) and also observed an enrichment of *Chitinophaga*, a genus from Chitinophagaceae family, following *B. sorokiniana* infection. However, this enrichment was significantly more pronounced under CMAA1741 + *B. sorokiniana* treatment (Log2FC = 2.08; Fig. 4C).

Similarly, the genus *Pseudomonas*, which includes the inoculated strain *P. inefficax* strain CMAA1741, was preferentially associated with this treatment, as indicated by both Random Forest and linear model analyses (p < 0.001, FDR < 0.005; Fig. 4C). Given its well-known biocontrol attributes, including antibiotic production (Baukova et al., 2024), siderophore synthesis (Grosse et al., 2023), and competitive root colonization (Sánchez-Gil et al., 2023), *Pseudomonas* likely played a central role in modulating disease outcomes (Yu et al., 2019, Sánchez-Gil et al., 2023). The increased abundance of *Pseudomonas* in natural soil suggests either successful establishment of the inoculant or stimulation of native strains. However, the lack of statistically significant disease suppression in this condition reinforces that high microbial diversity may constrain the efficacy of biocontrol agents, possibly by limiting their establishment or activity through microbial competition.

Nonetheless, the presence of *Pseudomonas* alongside other bacterial genera such as *Chitinophaga* and *Dyadobacter*, all of which were significantly more abundant in the CMAA1741 + *B. sorokiniana* treatment (Log2FC = 2.33; Fig. 4C), may reflect early-stage or context-dependent interactions with potential suppressive capacity. These genera are widely recognized as key players in disease-suppressive soils (Mendes et al., 2011, Carrión et al., 2019, Hussain et al., 2024, Khatri et al., 2024), and while suppression was not observed under high-diversity conditions, their relationship suggests the microbiome may have been shifting toward a suppressive configuration that could become functionally relevant over longer timescales or under different environmental contexts.

Taxonomic analysis of exclusive ASVs further revealed that the CMAA1741 + *B. sorokiniana* in natural soil harbored a larger number of Chitinophagaceae members compared to autoclaved soil (Fig. 4B). Natural soil contained seven *Chitinophagaceae* taxa, including *Flavisolibacter* (ASV-00092), *Olivibacter* (ASV-01082), *Terrimonas* (ASV-01033), *Niastella* (ASV-01284 and ASV-01297), *Taibaiella* (ASV-01308), and *Chitinophaga* (ASV-01693). In contrast, autoclaved soil contained only *Flavisolibacter metallilatus* (ASV-00166) and a species of (ASV-01106) (Fig. 5B). Although Random Forest analysis identified several bacterial taxa as potentially relevant in autoclaved soil during disease suppression (Fig. 5A), multiple regression detected a significant association only for the genus *Fluviicola*. This may give the interpretation that, in highly simplified communities, i.e. autoclaved soil, disease control was likely driven primarily by the direct antagonistic activity of *P. inefficax* strain CMAA1741, rather than by broader microbiome restructuring. In contrast, natural soil, inoculated with *P. inefficax* in the presence of the pathogen, exhibited significant enrichment of genera often associated with disease suppression (*Chitinophaga*, *Pseudomonas*, and *Dyadobacter*). This highlights the complexity of microbiome-mediated plant protection and suggests that, under high-diversity conditions, suppressive effects in conducive soils may depend on repeated pathogen exposure across multiple plant growth cycles, as previously observed by Costa et al. (2023).

Taken together, these results suggest that in low-diversity environments, *P. inefficax* strain CMAA1741 can act primarily through direct antagonism, whereas in more complex communities, where suppressive effects may require longer ecological stabilization or more synergistic interactions to manifest. These findings can indicate the context-dependent nature of disease suppression modulated by soil microbial diversity (Berg et al., 2021).

## 5 Conclusion

These results highlight the pivotal role of soil microbial diversity in shaping both pathogen establishment and the effectiveness of biocontrol strategies. *P. inefficax* strain CMAA1741 significantly suppressed *Bipolaris sorokiniana* in soils with reduced microbial diversity, conditions where the pathogen typically thrives and competition for niche space is minimal. Despite high pathogen abundance, CMAA1741 reduced disease severity in these simplified environments, emphasizing its potential as a biocontrol agent under low-diversity conditions. Conversely, high microbial diversity may limit the activity of the introduced biocontrol agent, illustrating the context-dependent nature of diversity in plant–microbe interactions. Although our controlled experimental system provided important insights into key microbiome-pathogen-inoculant dynamics, future field studies are crucial to assess how environmental variation and microbial complexity influence biocontrol performance. Long-term field trials and the development of synthetic microbial consortia (*SynComs*) may enhance the robustness and scalability of biocontrol approaches by leveraging synergistic interactions.

This study provides a framework for tailoring microbial inoculant strategies to specific soil conditions. Such precision could reduce the need for high inoculum loads and allow for spatially targeted applications, increasing cost-effectiveness and minimizing ecological disturbance. Ultimately, integrating microbial diversity assessments into disease management programs can enhance the sustainability and resilience of crop production systems.

## CRediT authorship contribution statement

**Caroline Sayuri Nishisaka:** Validation, Methodology, Formal analysis, Data curation, Conceptualization. **Hélio Danilo Quevedo:** Methodology, Data curation. **João Paulo Ventura:** Methodology, Data curation. **Fernando Dini Andreote:** Writing – review & editing, Conceptualization. **Tim H. Mauchline:** Writing – review & editing, Conceptualization. **Rodrigo Mendes:** Writing – review & editing, Writing – original draft, Validation, Supervision, Resources, Project administration, Methodology, Investigation, Funding acquisition, Data curation, Conceptualization.

## Declaration of competing interest

The authors declare that they have no known competing financial interests or personal relationships that could have appeared to influence the work reported in this paper.

## Data Availability

Extra data and metadata are included in supplementary material and are deposited at Zenodo (https://zenodo.org/records/14700441). The raw amplicon sequencing data are available at the NCBI under BioProject number PRJNA1111053. The soil sample access activity is registered in SISGen under the code A5EB05F. The genome of *Pseudomonas inefficax* CMAA1741 is accessible at NCBI GenBank under BioProject number PRJNA802578, with BioSample accession number SAMN25556618. Additional genome assembly details, SRA identifiers, and antiSMASH data are available at Zenodo (https://zenodo.org/records/10854821) (Nishisaka et al., 2024b).
