# Supplementary material for: Soil microbial diversity: A key factor in pathogen suppression and inoculant performance
Source: Geoderma. 2025 Aug;460:117444. doi: 10.1016/j.geoderma.2025.117444 (PMC12325084; doi:10.1016/j.geoderma.2025.117444)
Supplement: Supplementary Data 1 [file mmc1.pdf]

Supplementary Material

Soil Microbial Diversity: a key factor in pathogen suppression and inoculant performance

Caroline Sayuri Nishisaka[1,2], Hélio Danilo Quevedo[1,2], João Paulo Ventura[1,2], Fernando Dini Andreote[2], Tim H. Mauchline[3], Rodrigo Mendes[1]

[1] Embrapa Environment, Jaguariúna, SP, Brazil

[2] College of Agriculture "Luiz de Queiroz", University of São Paulo, Piracicaba, SP, Brazil

[3] Sustainable Soils and Crops, Rothamsted Research, Harpenden, Hertfordshire, UK

*Corresponding author: rodrigo.mendes@embrapa.br

This file contains:

Supplementary Figures: Figures S1 to S9

Supplementary Table: Tables S1 and S2

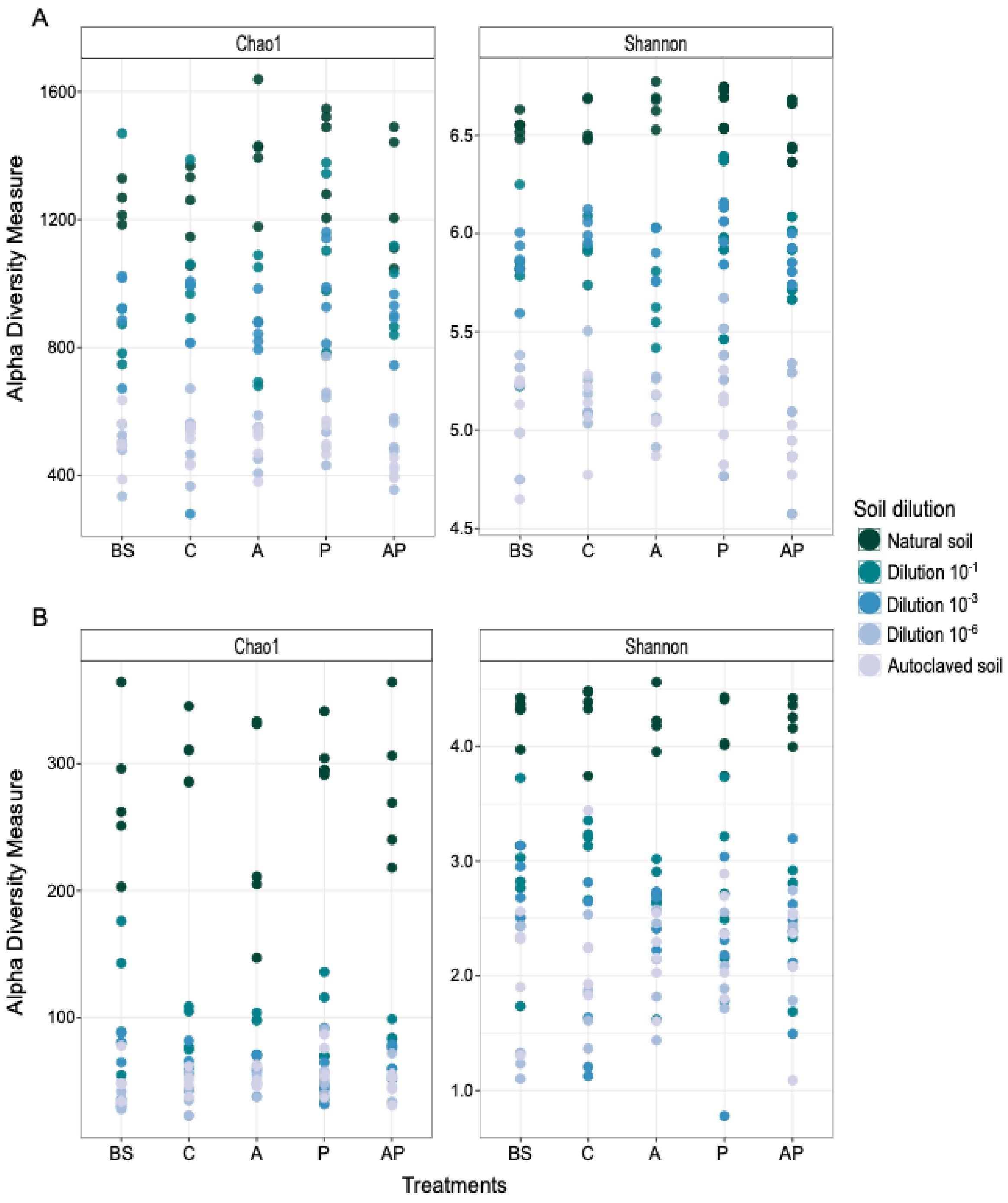

Figure S1. Alpha diversity of bacterial and fungal communities based on Chao1 and Shannon indices. **A** Bacterial alpha diversity in different soil dilutions under control (non-treated plants), CMAA1741 (plants inoculated with the antagonistic bacterium), *Bipolaris sorokiniana* (plants inoculated with the pathogen), and CMAA1741 + *B. sorokiniana* (plants inoculated with the antagonist and with the pathogen). **B** Fungal alpha diversity in different soil dilutions under the same treatments. Tukey HSD test ($P < 0.05$) was performed on the Shannon index to compare soil dilutions across different treatments. BS = bulk soil, C = control (non-treated plants), A = CMAA1741 (*P. inefficax* strain CMAA1741), P = *B. sorokiniana*, AP = CMAA1741 + *B. sorokiniana*.

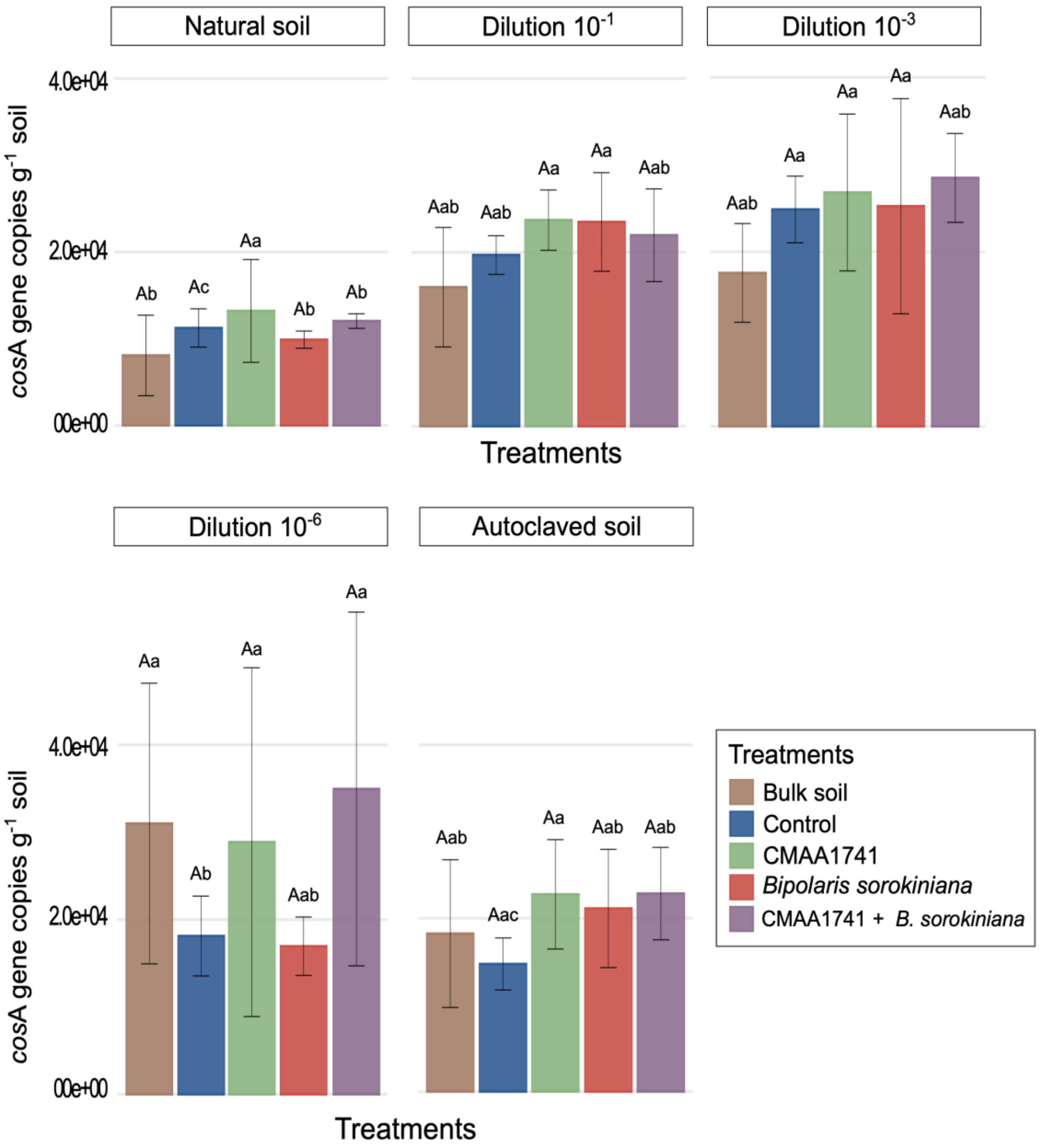

Figure S2. *Bipolaris sorokiniana* cosA gene quantification includes the following treatments: control (non-treated plants), CMAA1741 (plants inoculated with the antagonistic bacterium), *Bipolaris sorokiniana* (plants inoculated with the pathogen), and CMAA1741 + *B. sorokiniana* (plants inoculated with the antagonist and with the pathogen), in five different soil dilutions. Mean comparisons were conducted using the Tukey test ($P < 0.05$). Uppercase letters denote comparisons between treatments within the same soil diversity, and lowercase letters indicate comparisons of the same treatment across different soil dilutions.

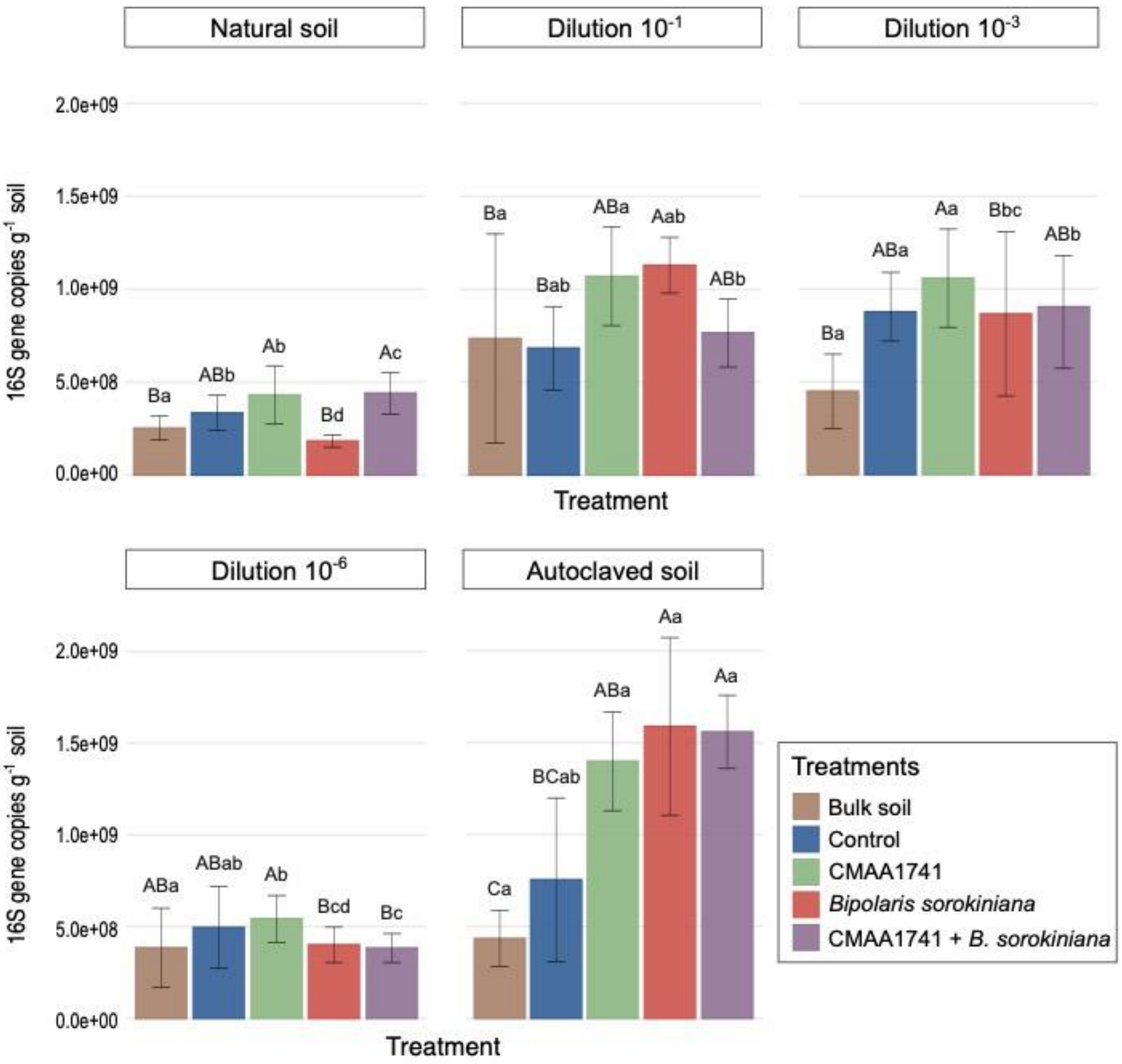

Figure S3. Quantification of bacterial 16S rRNA genes under control (water), *Pseudomonas inefficax* strain CMAA1741 (CMAA1741), *Bipolaris sorokiniana*, and CMAA1741 + *B. sorokiniana* treatments across different soil dilutions. Mean comparisons were conducted using the Tukey test ($P < 0.05$) for soil dilution comparisons within treatments.

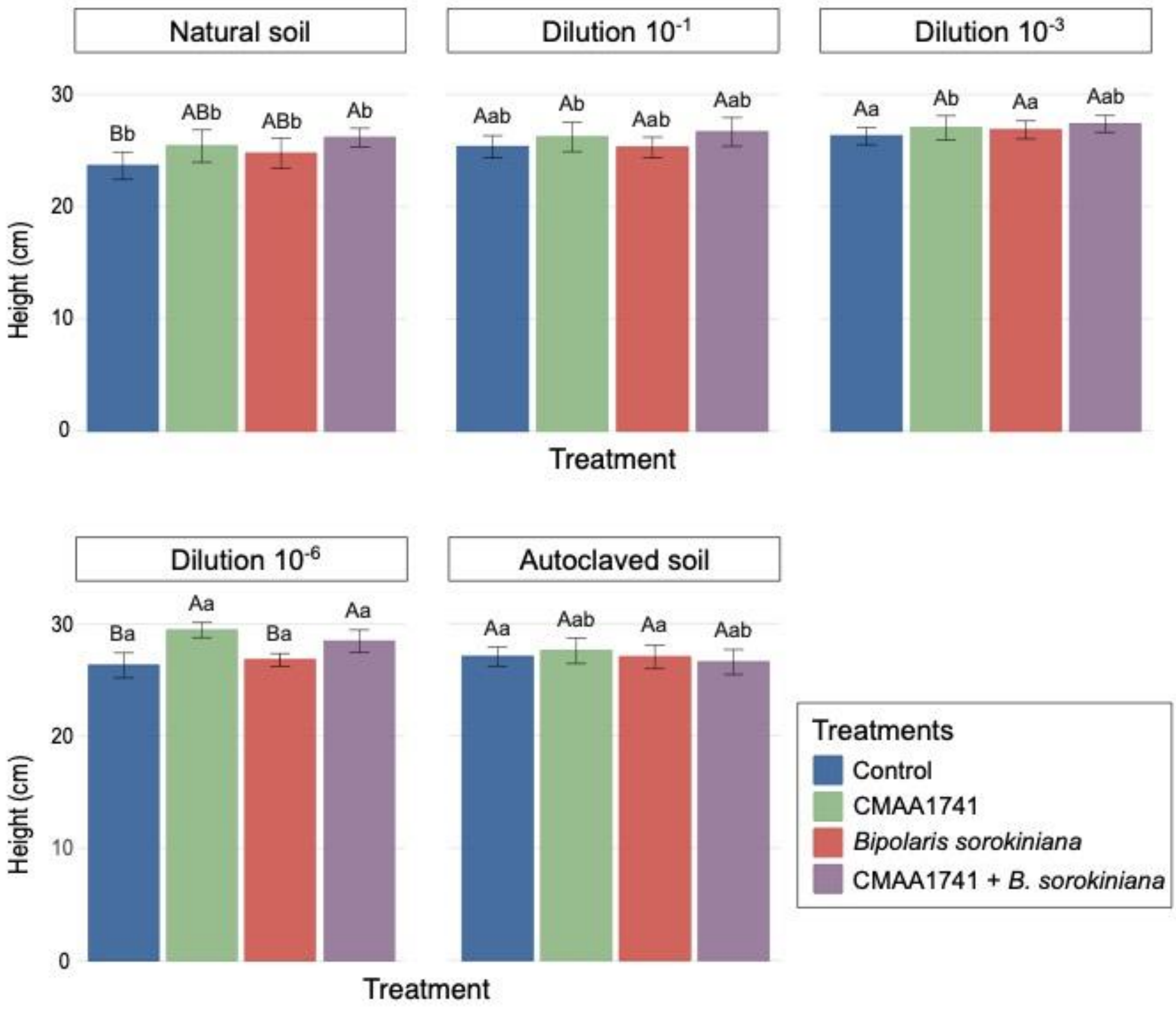

Figure S4. Plant heights under different soil diversities across control (water), *Pseudomonas inefficax* strain CMAA1741 (CMAA1741), *Bipolaris sorokiniana*, and CMAA1741 + *B. sorokiniana* treatments. Mean comparisons were conducted using the Tukey test ($P < 0.05$). Uppercase letters denote comparisons between treatments within the same soil diversity, while lowercase letters indicate comparisons of the same treatment across different soil diversities.

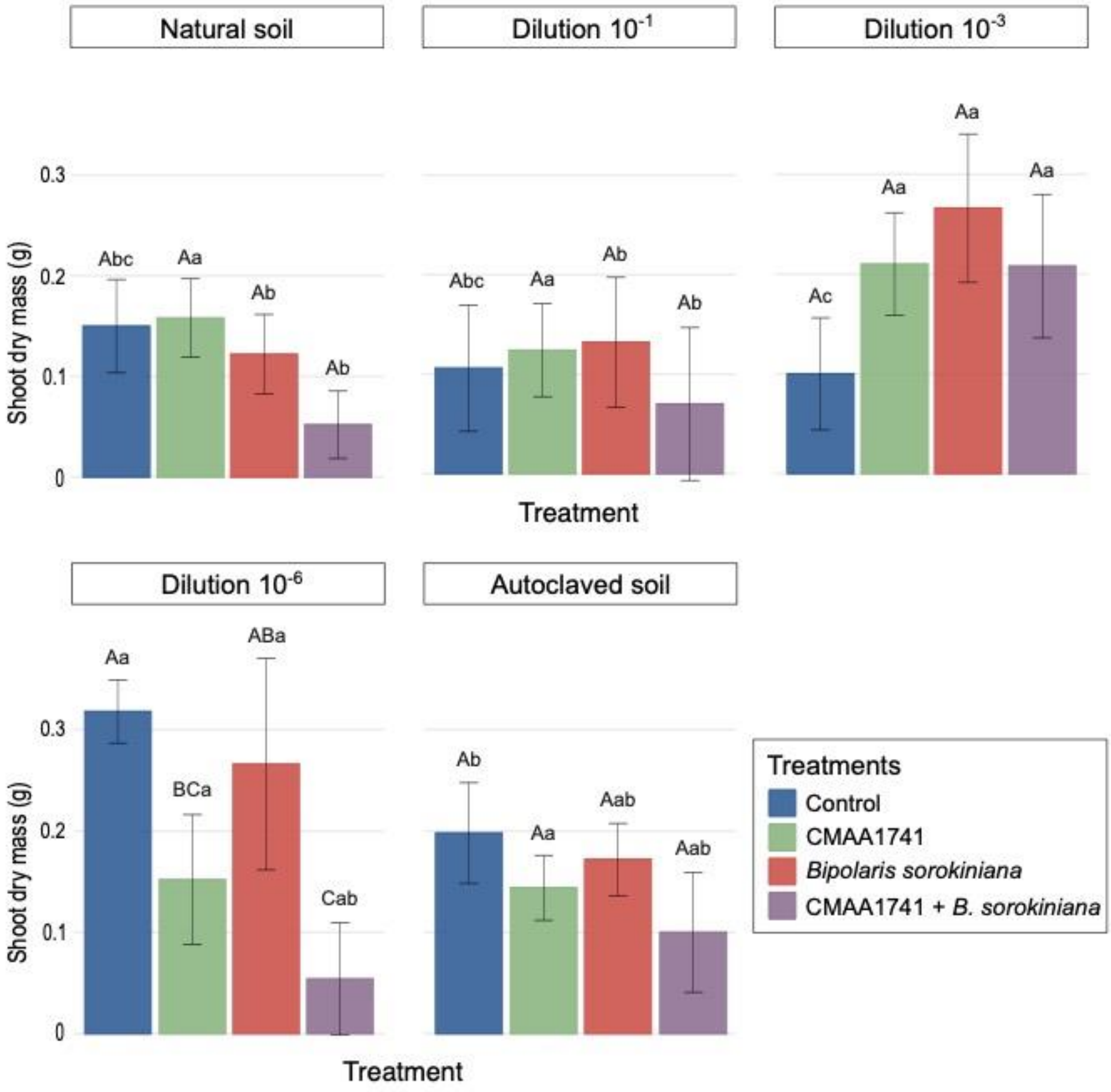

Figure S5. Plant shoot dry masses across different soil diversities under control (water), *Pseudomonas inefficax* strain CMAA1741 (CMAA1741), *Bipolaris sorokiniana*, and CMAA1741 + *B. sorokiniana* treatments. Mean comparisons were conducted using the Tukey test ($P < 0.05$). Uppercase letters denote comparisons between treatments within the same soil diversity, while lowercase letters indicate comparisons of the same treatment across different soil diversities.

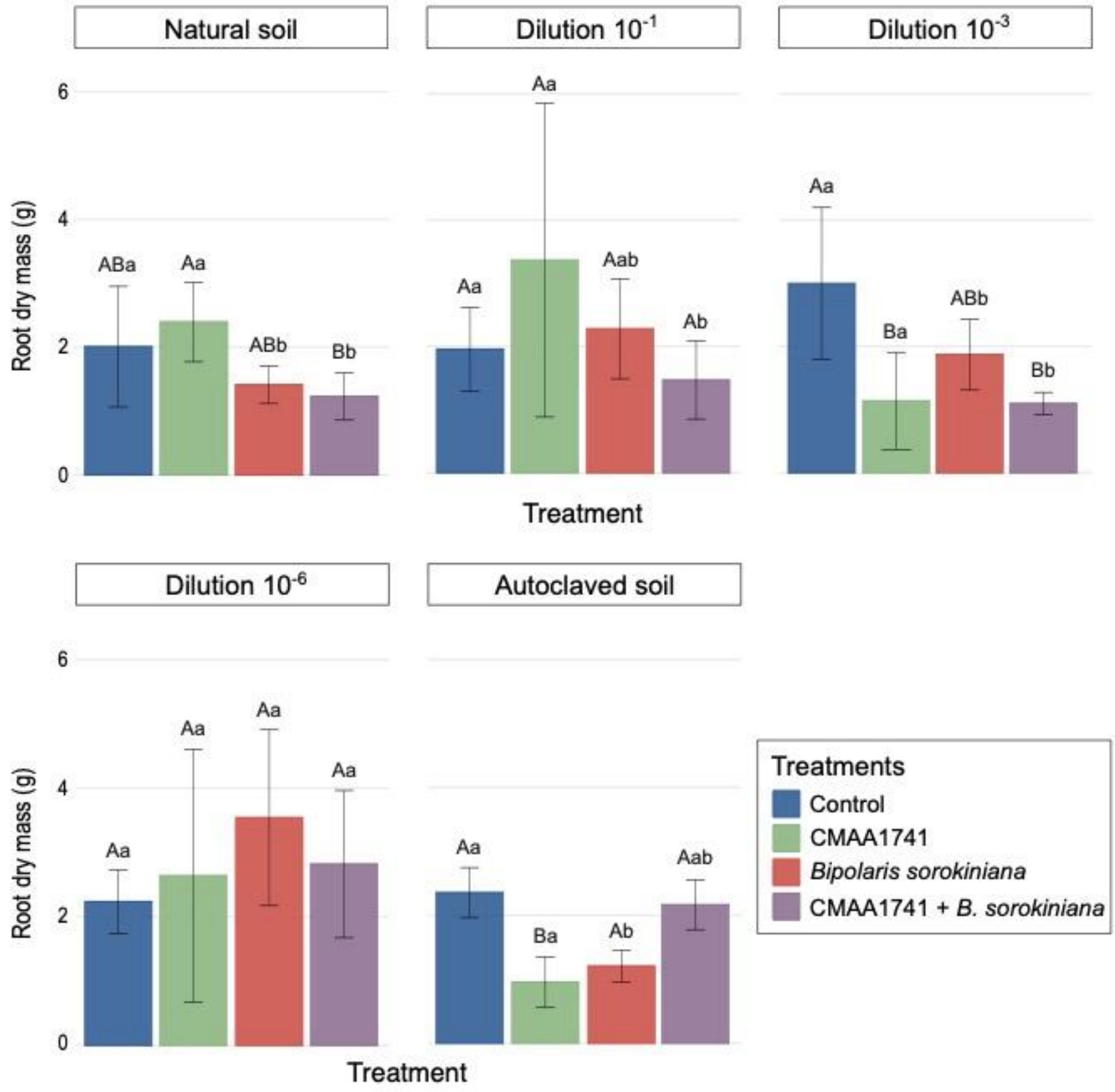

Figure S6. Plant root dry masses across different soil diversities under control (water), *Pseudomonas inefficax* strain CMAA1741 (CMAA1741), *Bipolaris sorokiniana*, and CMAA1741 + *B. sorokiniana* treatments. Mean comparisons were conducted using the Tukey test ($P < 0.05$). Uppercase letters denote comparisons between treatments within the same soil diversity, while lowercase letters indicate comparisons of the same treatment across different soil diversities.

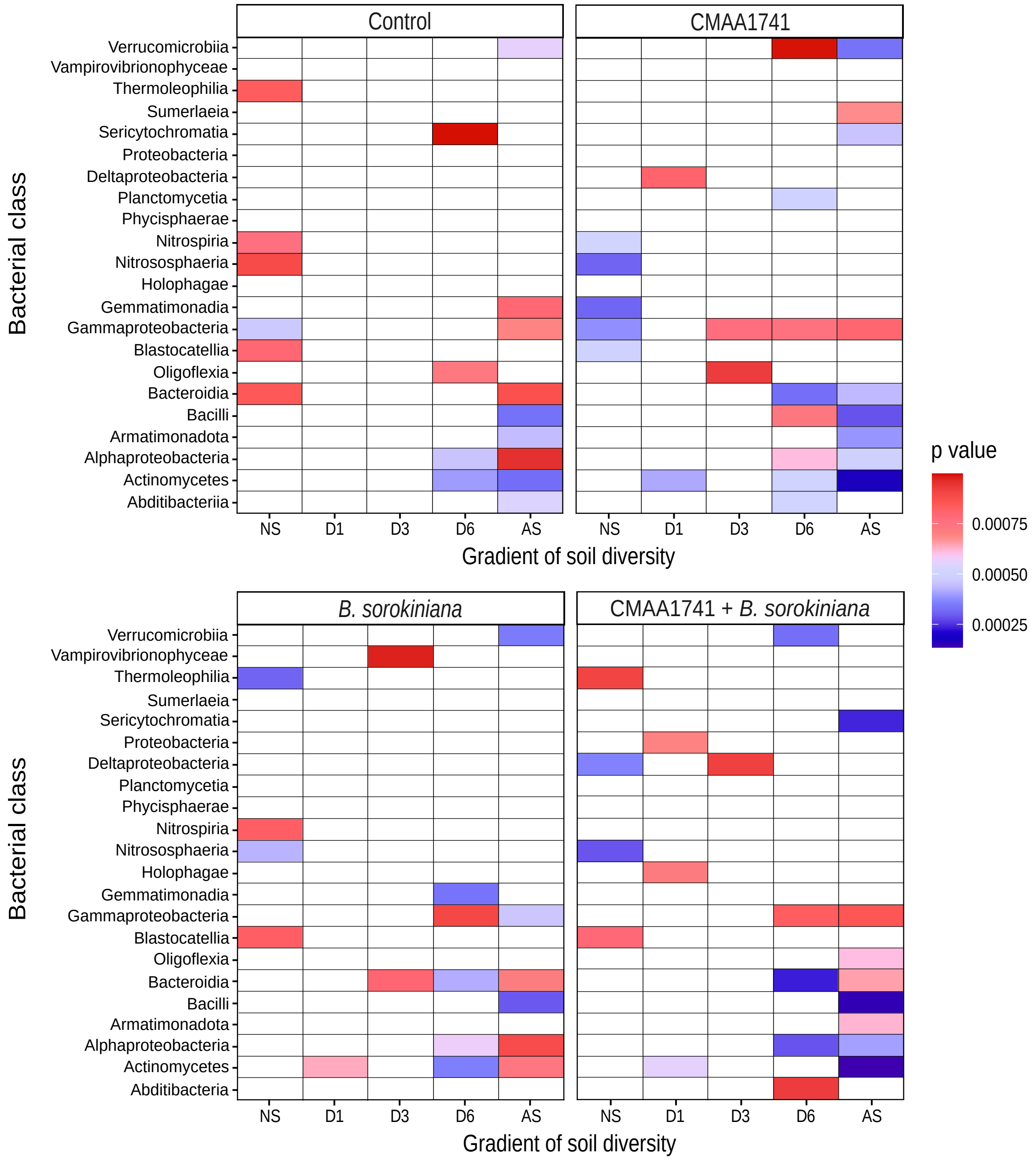

Figure S7. Differential abundance of bacterial classes across different soil dilutions for each treatment. Abbreviations: NS = natural soil, D1 = dilution at 10^{-1}, D3 = dilution at 10^{-3}, D6 = dilution at 10^{-6}, AS = autoclaved soil.

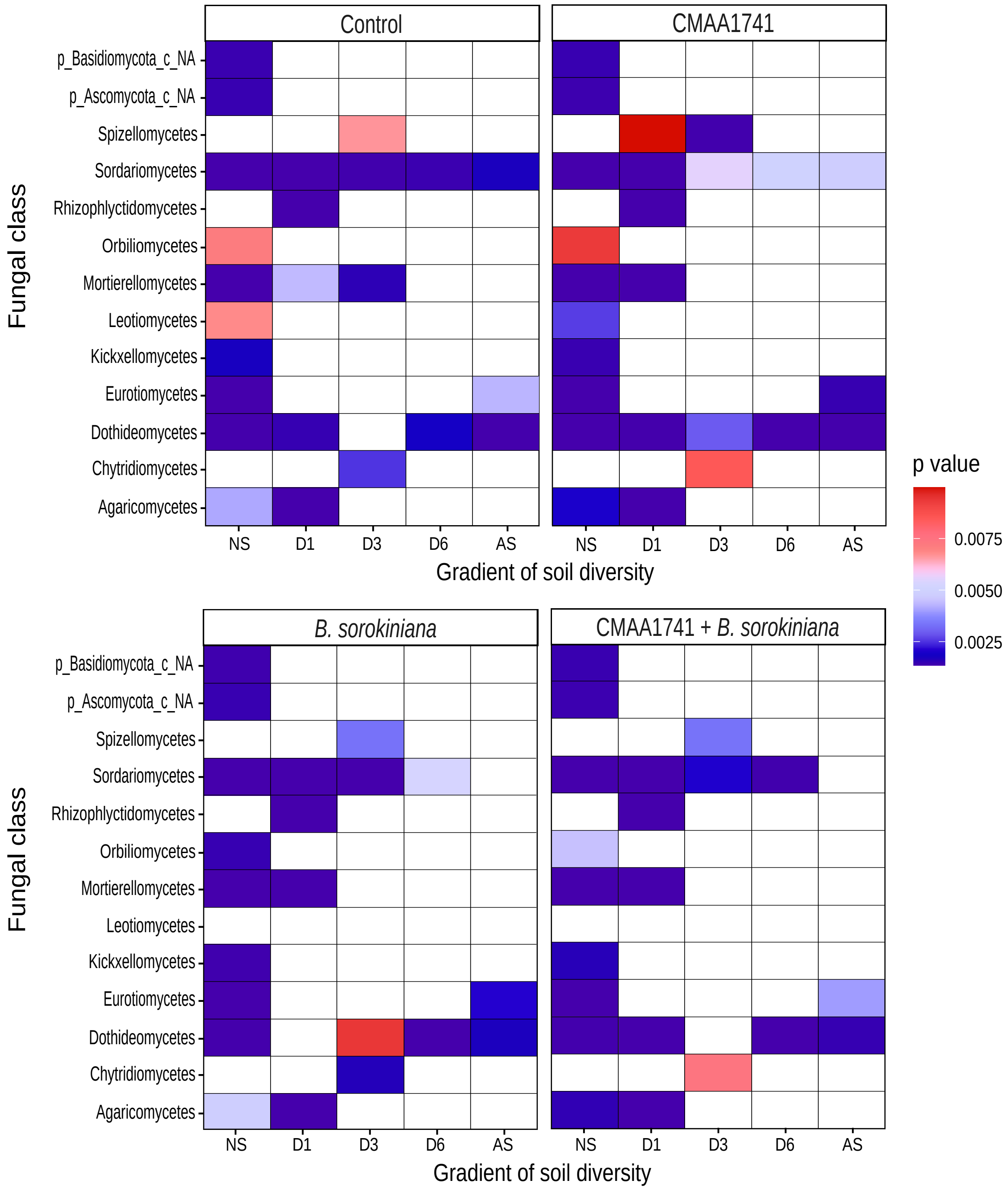

Figure S8. Differential abundance of fungal classes across different soil dilutions for each treatment. Abbreviations: NS = natural soil, D1 = dilution at 10^{-1}, D3 = dilution at 10^{-3}, D6 = dilution at 10^{-6}, AS = autoclaved soil.

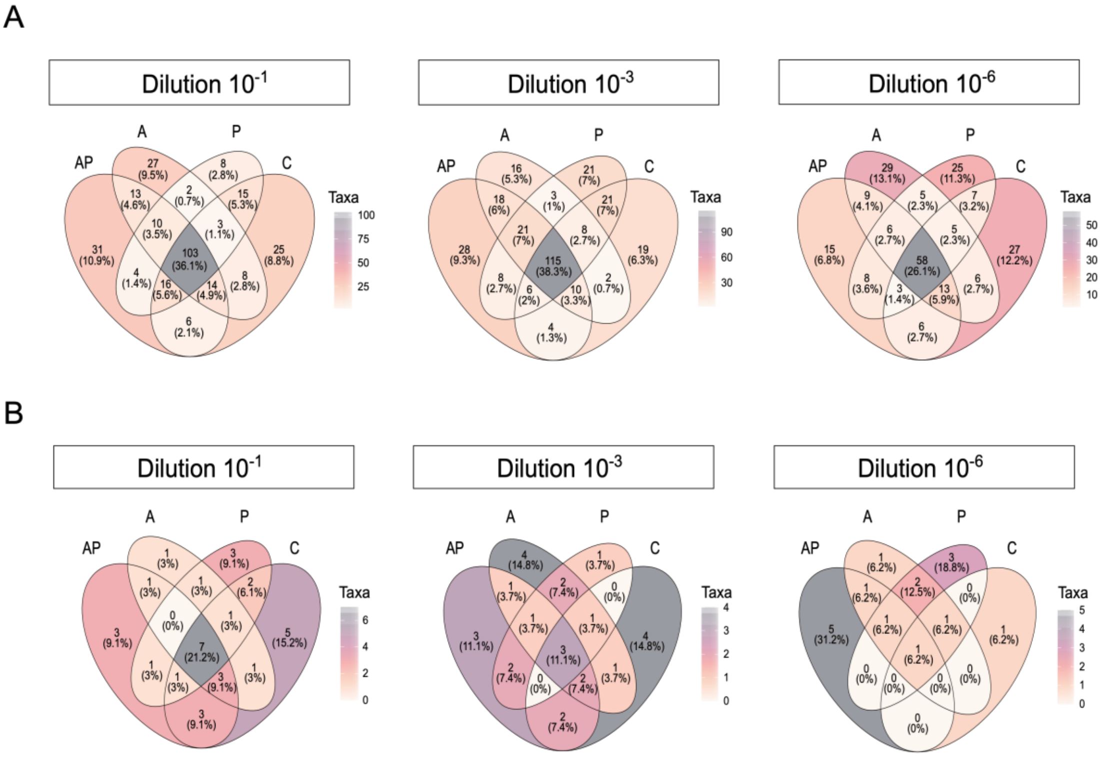

Figure S9. Venn diagram of unique and shared ASVs among treatments in different soil diversities. **A** Venn diagram highlighting exclusive and shared bacterial taxa among treatments under dilution 10^{-1}, 10^{-4}, and 10^{-6}, considering core microbiome detection in at least 90% of samples and a prevalence of 50%. **B** Venn diagram highlighting exclusive and shared fungal taxa among treatments under dilution 10^{-1}, 10^{-4}, and 10^{-6}, considering core microbiome detection in at least 90% of samples and a prevalence of 50%. C = control (non-treated plants); A = CMAA1741 (*Pseudomonas inefficax* strain CMAA1741); P = *Bipolaris sorokiniana*; AP = CMAA1741 + *B. sorokiniana*

Table S1 Physical-chemical properties of natural and autoclaved soil

Properties	Unit	Soil sample	
		**Natural Soil**	**Autoclaved soil**
Soil chemical properties			
OM	g/dm^3	24	24
pH	-	5.3	5.1
H + Al	mmolc/dm^3	13	11
P	mg/dm^3	47	42
K	mmolc/dm^3	2.9	3
Ca		57	56
Mg		26	28
Na		-	-
SB		85.9	87
CEC		98.9	98
Al		0	0
V%	%	87	89
S	mg/dm^3	6	11
B		0.32	0.37
Cu		0.6	0.5
Fe		26	21
Mn		6.9	15.3
Zn		1.3	1.2
EC	dS/m	0.2	0.3
N	g/kg^{-1}	1.3	1.3
Cl	mg/dm^3	55.8	51.6
Cd		<0,1	<0,1
Cr		<0,1	<0,1
Pb		0.75	0.69
Ni		<0,01	<0,01
Soil physical properties			
Soil density	mg m^{-3}	1.2	1.19
Soil humidity	FC g^3	0.3515	0.3025
	PWP g^{-3}	0.2059	0.194
Clay	%	45.3	46.4
	g/kg	453	464
Silt	%	6.4	6.6
	g/kg	64	66
Total sand	%	48.3	47
	g/kg	483	470
Textural classification	-	Sandy-clay	Sandy-clay

Soil chemical properties - **OM** = Organic matter. pH was measured using 0.01 M CaCl2; H+Al content was measured using the SMP-buffer method; P, K, Ca, and Mg content were measured using resin extraction; Na content was measured using Al acetate at pH 7; **SB** = Sum of bases; **CEC** = Cation exchange capacity; Al content was measured using potassium chloride extraction; S content was measured using calcium phosphate extraction; B content was measured using hot water extraction; Cu, Fe, Mn, Zn, Cl, Cd, Cr, Pb, and Ni content were measured using DTPA extraction; **EC** = Electrical conductivity was measured using a 1:1 water extraction; **N** content was measured using the Kjeldahl method.

Soil physical properties – Soil density was obtained by deformed sample; soil moisture was obtained with two methods: field capacity (FC) and permanent wilting point (PWP); granulometry measurement was obtained by pipette method (Clay < 0.002 mm, silt = 0.053 – 0.002 mm, and total sand = 2.00 – 0.053 mm).

Table S2 Pairwise Adonis test of treatments in different soil dilutions

Treatments			Natural soil	Dilution 10^{-1}	Dilution 10^{-3}	Dilution 10^{-6}	Autoclaved soil
			**P value**				
Bacterial community							
Control	*vs.*	CMAA1741	0.011*	0.013*	0.011*	0.008*	0.120
Control	*vs.*	*B. sorokiniana*	0.010*	0.467	0.320	0.037*	0.016*
Control	*vs.*	CMAA1741 + *B. sorokiniana*	0.013*	0.004*	0.013*	0.015*	0.014*
CMAA1741	*vs.*	*B. sorokiniana*	0.008*	0.034*	0.008*	0.020*	0.174
CMAA1741	*vs.*	CMAA1741 + *B. sorokiniana*	0.024*	0.075	0.422	0.041*	0.345
B. sorokiniana	*vs.*	CMAA1741 + *B. sorokiniana*	0.011*	0.042*	0.010*	0.130	0.330
Fungal community							
Control	*vs.*	CMAA1741	0.160*	0.057	0.557	0.094	0.026*
Control	*vs.*	*B. sorokiniana*	0.009*	0.207	0.197	0.061	0.007*
Control	*vs.*	CMAA1741 + *B. sorokiniana*	0.006*	0.234	0.301	0.025*	0.095
CMAA1741	*vs.*	*B. sorokiniana*	0.011*	0.162	0.215	0.270	0.036*
CMAA1741	*vs.*	CMAA1741 + *B. sorokiniana*	0.063	0.301	0.263	0.029*	0.013*
B. sorokiniana	*vs.*	CMAA1741 + *B. sorokiniana*	0.015*	0.397	0.453	0.106	0.060

(*) Asterisk means a significant difference according to the pairwise Adonis test ($P<0.05$).
